# Assessment of a Multiplex Serological Test for the Diagnosis of Prosthetic Joint Infection: a Prospective Multicentre Study

**DOI:** 10.7150/jbji.42076

**Published:** 2020-03-30

**Authors:** Pascale Bémer, Céline Bourigault, Anne Jolivet-Gougeon, Chloé Plouzeau-Jayle, Carole Lemarie, Rachel Chenouard, Anne-Sophie Valentin, Sandra Bourdon, Anne-Gaëlle Leroy, Stéphane Corvec, The Centre de Référence des Infections Ostéo-articulaires du Grand Ouest (CRIOGO) Study Team

**Affiliations:** 1 Bacteriology Department, CHU Nantes, Nantes Université, Nantes, France.; 2 Bacteriology and Infection Control Department, CHU Nantes, Nantes Université, Nantes, France.; 3 Bacteriology Department, INSERM, CHU Rennes, Rennes Université, Rennes, France.; 4 Bacteriology Department, CHU Poitiers, Poitiers Université, Poitiers, France.; 5 Bacteriology Department, CHU Angers, Angers Université, Angers, France.; 6 Bacteriology Department, CHU Tours, Tours Université, Tours, France.; 7 Bacteriology Department, CH La Roche/Yon, La Roche/Yon, France.

**Keywords:** prosthetic joint infection, serological diagnosis, anti-staphylococcal antibodies, sensitivity, specificity, negative predictive value

## Abstract

**Introduction:** The diagnosis of prosthetic joint infections (PJIs) can be difficult in the chronic stage and is based on clinical and paraclinical evidence. A minimally invasive serological test against the main pathogens encountered during PJI would distinguish PJI from mechanical loosening.

**Methods:** We performed a prospective, multicentre, cross-sectional study to assess the contribution of serology in the diagnosis of PJI. Over a 2-year period, all patients undergoing prosthesis revision were included in the study. A C-reactive protein assay and a serological test specifically designed against 5 bacterial species (*Staphylococcus aureus*, *S. epidermidis*, *S. lugdunensis, Streptococcus agalactiae*, *Cutibacterium acnes*) were performed preoperatively. Five samples per patient were taken intraoperatively during surgery. The diagnosis of PJI was based on clinical and bacteriological criteria according to guidelines.

**Results:** Between November 2015 and November 2017, 115 patients were included, 49 for a chronic PJI and 66 for a mechanical problem. Among patients with PJI, a sinus tract was observed in 32.6% and a C-reactive protein level ≥10 mg/L in 74.5%. The PJI was monomicrobial in 43 cases (targeted staphylococci, 24; *S. agalactiae*, 1; *C. acnes*, 2; others, 16), and polymicrobial in 6 cases (12.2%). Sensitivity, specificity, positive predictive value and negative predictive value were 75.0%, 82.1%, 58.3% and 90.8%, respectively, for targeted staphylococci. Specificity/negative predictive value was 97.3%/100% for *S. agalactiae* and 83.8% /96.9% for *C. acnes*.

**Conclusions:** The serological tests are insufficient to affirm the diagnosis of PJI for the targeted bacteria. Nevertheless, the excellent NPV may help clinicians to exclude PJI.

## Introduction

Prosthetic joint infections (PJIs) may require one or more surgical procedures associated with antibiotic therapy and can lead to significant functional disability, risk of amputation or death 1,2. Chronic PJI, occurring more than 3 months after prosthesis implantation, account for most cases 2,3. The clinical signs are non-specific when they include pain and inflammation in the absence of fever. The cornerstone for the diagnosis remains bacteriological culture of pre- and/or perioperative samples, which is considered as a major criterion by the Infectious Diseases Society of America (IDSA), the International Consensus on Orthopaedic Infections, and other European learned societies 4-8.

When the diagnosis of PJI is confirmed by culture, the bacteria most often isolated from monomicrobial PJIs are *Staphylococcus aureus* and coagulase-negative staphylococci (isolated in 60% - 65% of PJIs), followed by streptococci, enterococci, *Enterobacteriaceae* and anaerobes (mainly *Cutibacterium acnes*, isolated in about 8% of PJIs) 1,2,3. Aero-anaerobic polymicrobial infections account for about 15% of PJIs 2,3.

The BJI InoPlex® (Diaxonhit, Paris, France) test is the first multiplex serological test proposed for the diagnosis of PJI to provide a preoperative diagnostic orientation. A selection of recombinant antigens corresponding to the bacteria most often isolated in PJIs has been targeted. Three species of staphylococci (*S. aureus*, *S. epidermidis*, *S. lugdunensis*), *Streptococcus agalactiae* and *C. acnes* are the main anaerobic species isolated from PJIs. The technological innovation, developed by Diaxonhit allows a simple, quick and inexpensive diagnosis from a blood sample. The test does not provide information on the time of infection by detecting only IgGs and not IgMs, and can theoretically be the cause of negative results if it is performed too early in the course of infection. Patients with acute PJI should be excluded from testing according to the supplier's recommendations.

Two recent studies have evaluated its contribution to the diagnosis of chronic PJIs 9,10. The original study for the development and the evaluation of the test included 455 patients in 2 referral centres for complex osteoarticular infections, 176 PJIs and 279 uninfected patients 9. The second prospective, single-centre study included 71 patients, 26 with aseptic failure and 45 with PJI in another referral centre for complex osteoarticular infections 10. The preliminary prospective study showed variable performance for detection, with a sensitivity and specificity of 72.3% and 80.7%, respectively for staphylococci, 75% and 92.6%, respectively, for *S. agalactiae*, and 38.5% and 84.8%, respectively for *C. acnes* 9. The results reported in the second study for the sensitivity, specificity, positive and negative predictive values were 81.8%, 95.4%, 97.3% and 72.4%, respectively, for all patients and 87.5%, 93.5%, 94.6% and 85.3%, respectively, for staphylococcal infections 10.

Our study assesses the contribution of the BJI InoPlex® test for the routine diagnosis of chronic PJI in a blinded, prospective and multicentre manner. The interregional organization of our reference centre for the management of osteoarticular infections enabled us to recruit patients in 7 different centres 3.

## Materials and Methods

### Study design

This study was designed as a multicentre, prospective, observational study of adult patients suspected to have PJI. The study protocol was approved by the institutional review board and ethics committee (RC15_0439). Informed consent was obtained from each patient before inclusion.

### Evaluation criteria

The main objective of the study was to evaluate the performances (sensitivity, specificity, positive and negative predictive values) of the serological test for the diagnosis of PJI. Our standard of comparison was the results of cultures of pre- and/or perioperative samples.

### Inclusion criteria

Adult men or women who were candidates for a prosthesis revision for a mechanical problem (signs of wear, radiological loosening, poor positioning) or had a suspected chronic infection (fistula near the prosthesis, pain and/or radiological loosening at least 3 months since prosthesis implantation) were included. Patients were included either on the advice of the surgeon preoperatively or in a weekly multidisciplinary consultation meeting in each centre.

### Exclusion criteria

Patients less than 18 years of age and patients with acute PJI (with symptoms for less than 3 months) were excluded from testing.

### Definition of PJI

According to the IDSA, International Consensus on Orthopaedic Infections and European Bone and Joint Infection Society guidelines, definitive evidence of PJI is a sinus tract in communication with the prosthesis and/or 2 or more perioperative cultures or a combination of preoperative and perioperative cultures that yield the same organism 2,4,5,7.

### Course of the study

#### The evaluation parameters

Case report forms were created for collection of the following data for each patient: patient characteristics, arthroplasty localization, date of prosthesis placement, presence of fistula and antibiotic treatment in the 15 days before surgery, C-reactive protein level (mg/L), and antibodies produced in response to specific staphylococci, *C. acnes* and *S. agalactiae* antigens*.*

#### Bacteriological methods

Cultures of periprosthetic tissue samples were performed in each centre following a standardized protocol. For each patient, 5 perioperative specimens were collected in sterile vials with different surgical instruments. They were milled (MM401 Retsch) and cultured on appropriate aero-anaerobic media incubated at 37°C. Validation of the results occurred after 14 days of culture as recommended. Isolated bacteria were identified according to standard laboratory procedures 3,11.

#### The biological parameters

CRP assays were performed in each participating centre. Serological analysis was performed on serum taken during the preoperative assessment, centrifuged and stored at -80°C until tested. Each participating centre sent the frozen sera tubes to the investigating laboratory.

#### Principle of the BJI Inoplex test

This first serological test analyses the immune response in PJI. Sixteen antigens are attached to the surface of microbeads (8 for *S. aureus*, *S. epidermidis* and *S. lugdunensis*, 4 for *S. agalactiae*, and 4 for *C. acnes*). This is a minimally invasive test and the response is available in about 2 h. The protocol is as follows: microbeads coated with the selected antigens were incubated with the patient's serum (10 μL of test sample in a single well). Negative and positive controls, as well as calibrators, were included in each series. After 2 incubation steps of 30 min each at room temperature, phycoerythrin-conjugated goat antihuman IgG was applied to the wells. Then, after 2 washing steps of 2 min each, the analysis was performed on the MAGPIX (Luminex) PLC. The interpretation, using the Diaxonhit software, combined the intensity of the reactions obtained and the number of positive antigens for each family. The qualitative results provided were positive, negative or indeterminate in favour of or against an infection. In cases where the test was positive, indeterminate or invalid, it was repeated once. If the test was again indeterminate, it was excluded from the analysis. If the serum was positive for all the antigens tested (non-specific cross-positive reactions), it was considered invalid and excluded from the analysis according to the supplier's recommendations.

#### Measures taken to reduce and avoid study biases

Serological results were interpreted blindly from classification of patients for potential infection, bacteriological results and CRP values.

### Statistical analysis

Descriptive analyses were conducted to compare infected and uninfected patients. Characteristics were compared using the chi-squared test and the Student t test. The diagnostic values of the test were measured for the entire population. Indeterminate results and positive cross-reactions were excluded from analyses. *P* values <0.05 were considered statistically significant. All statistical analyses were done using STATA 12.1 (Stata Corporation, College Station, Texas, USA).

## Results

### The study population

**Figure 1:**
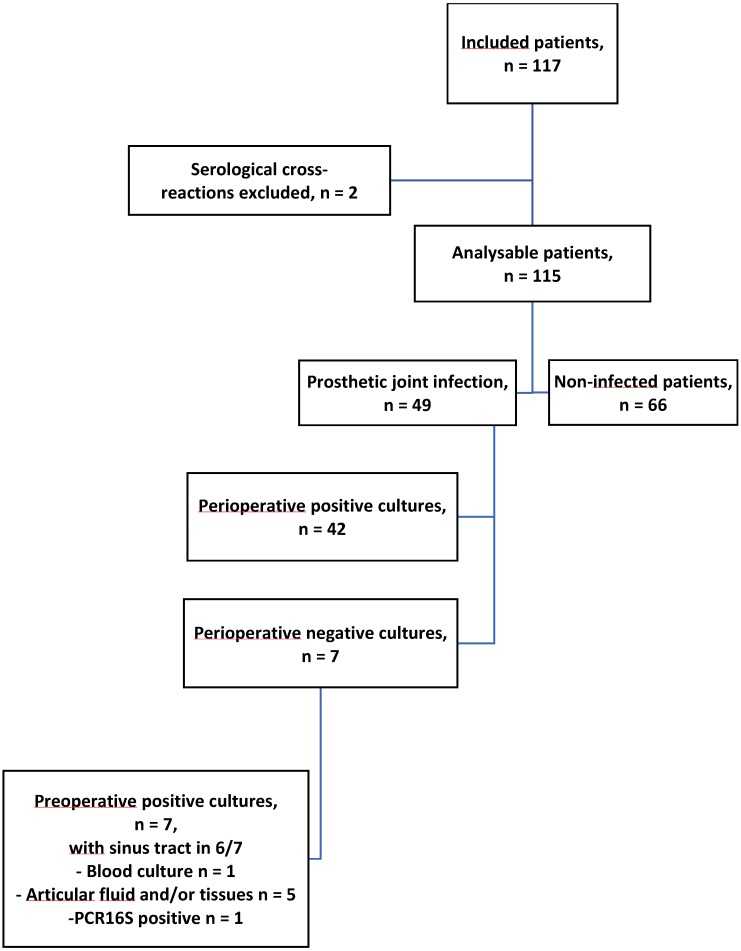
Flow chart of the study.

Between November 5, 2015, and November 28, 2017, 117 patients were included in the study (56 in Nantes, 40 in Rennes, 12 in Poitiers, 4 in Angers, 3 in Tours and 2 in La Roche/Yon), 50 for suspicion of PJI and 67 for a mechanical problem (Fig. 1). Two patients with serological cross-reactions were excluded from the analysis. Overall, 115 patients were included in the study: 49 patients with suspicion of PJI and 66 patients with a mechanical problem. The baseline demographic and clinical details are summarized in Table 1. The median age was 69 years (interquartile range, 63-78 years), and 49.6% were men. The affected joint was a hip in 67.0%, a knee in 29.6% and a shoulder in 3.5%. The characteristics of the infected and non-infected patients were not significantly different for age, sex, immunosuppressive therapy and rheumatoid arthritis. Infected patients were more likely to have had recent or ongoing antibiotic therapy (*p*= 0.033), been hospitalized in the previous 3 months (*p*= 0.003), had a shorter time since prosthesis placement (*p*= 0.001), have a sinus tract (*p*<0.0001) and an increased CRP level (*p*< 0.001). None of the 66 uninfected patients had a sinus tract, and 60 had perioperative samples sterile in culture. For 6 patients, 1 of 5 samples was positive for a skin flora bacterium; for these 6 patients, the serological test was negative for all the antigens tested.

Among the 49 infected patients, 42 had positive perioperative cultures, and 7 patients treated at the time of surgery had perioperative negative cultures (Fig. 1). Among these 7 patients, 1 patient was positive for *S. aureus* by 16S polymerase chain reaction, and 3 patients were found to have *S. aureus* (n = 2) and *S. epidermidis* (n = 1) preoperatively; the BJI Inoplex test detected anti-staphylococcal antibodies in these 4 cases. The 3 last patients had preoperative samples positive for *S. capitis* (n = 1), *Mycobacterium bovis* (n = 1) and *Streptococcus dysgalactiae* (n = 1). The test did not detect anti-staphylococcal antibodies.

### Microbial species in the preoperative or perioperative samples

Among the 49 PJIs, the infection was monomicrobial in 43 cases and polymicrobial in 6 cases (Table 2). Briefly, a *Staphylococcus* strain was involved in 32 PJIs with a species targeted by the serological test in 24 cases (*S. aureus* 13, *S. epidermidis* 10, *S. lugdunensis* 1), an *Enterococcus* or *Streptococcus* in 6 cases, including 1 identified with *S. agalactiae*; *C. acnes* was isolated in 2 PJIs. Among the 6 cases of polymicrobial infections, at least 1 of the species of bacteria was targeted by the serological test in 4 cases.

### Serological test performance by targeted species (Table 3)

The test was repeated 29 times, 11 times due to an indeterminate result and 18 times for an invalid result. Six results remained indeterminate and were excluded from the analysis, 3 for an anti-staphylococcal response and 3 for an anti-*S. agalactiae* response.

The sensitivity, specificity, positive (PPV) and negative (NPV) predictive values of the antibody response to target staphylococcal species were 75.0%, 82.1%, 58.3% and 90.8% respectively. Concerning *S. agalactiae* and *C. acnes* species, sensitivity and PPV were not evaluable given the low number of PJIs documented for these bacteria. Specificity and NPV were 97.3% and 100% for *S. agalactiae*, respectively, and 83.8% and 96.9% for *C. acnes*, respectively. Among the 7 treated patients with perioperative negative cultures, the BJI InoPlex® test detected anti-staphylococcal antibodies in the 4 cases of staphylococcal PJI diagnosed preoperatively.

### Performance of the serological test in association with the CRP value

The performance of the test in association with the CRP level was performed only for staphylococcal targets. The PPV of a positive serological test for targeted staphylococci associated with CRP >10 mg/L was 85.71%. The NPV of a negative serological test for targeted staphylococci associated with CRP <10 mg/L was 83.05%.

## Discussion

Our study has several methodological advantages: (a) it was conducted prospectively in 7 centres specialized in the management of complex osteoarticular infections, (b) patients with clinical signs of acute infection were not included in the study, (c) the serological results were interpreted blindly from the bacteriological and CRP results, and (d) this study included a relatively close number of infected and uninfected patients, making it possible to evaluate the PPVs and NPVs of the test.

Not surprisingly, the population analysed in our study included as many men as women, mostly with total hip and knee replacement. Patients were distributed evenly between PJI and mechanical problems. All PJIs were chronic, with the presence of a sinus tract in a third of cases and a significantly increased CRP level, which reinforces the importance of measuring CRP in the diagnosis of PJI 7-10.

The BJI InoPlex® serological test is run against only 5 of the most common bacterial species found in PJIs. The principal findings of our study showed sensitivity, specificity, PPV and NPV of 75.0%, 82.1%, 58.3% and 90.8% for targeted staphylococci, respectively, and specificity and NPV of 97.3% and 100% for *S. agalactiae*, and 83.8% and 96.9% for *C. acnes*. The sensitivity and PPV of the test for both *S. agalactiae* and *C. acnes* species could unfortunately not be evaluated in our study, given the low number of infections with these bacteria. The specificity of the test against *S. agalactiae* was excellent, due to the low number of false-positive reactions. In contrast, as observed for the targeted staphylococci, a high number of false-positive reactions against *C. acnes* was observed among uninfected patients (17 of 66), which significantly reduced the SP to 84.6%.

The sensitivity and specificity of the detection of anti-staphylococcal antibodies in our study are close to those reported by Marmor et al. (72.3% and 80.7%, respectively) and lower than those reported by De Seynes et al. (87.5% and 93.5%, respectively) 9,10. The specificity is notably lower than that reported by De Seynes et al. with the high number of positive serological reactions among uninfected patients (18.1%) in our study. It follows that the PPV (probability that the patient has a staphylococcal infection when the test is positive) is only 57.1% in our study, whereas it is 94.6% in the study by De Seynes et al. 10. In our study, we have demonstrated positive anti-staphylococcal serology in patients treated for *S. aureus* PJI, even though the number of patients concerned is small.

Optimization of the bacteriological diagnosis in our study is important. The high number of samples required, grinding and seeding the ground material in several aero-anaerobic culture media guarantee the high sensitivity of the diagnostic methods used. Thus, when at least 5 samples from uninfected patients remain sterile in culture after 14 days of incubation in the absence of antibiotic treatment, the anti-staphylococcal positive serological reactions may be false-positive results.

The best performance of the test against the target bacteria was the NPV (90.7%, 96.9% and 100% for the 3 species of target staphylococci, *C. acnes* and *S. agalactiae*, respectively). However, even if these 3 species of staphylococci are responsible for more than half of PJIs, the NPV cannot be extrapolated to all bacteria isolated from PJIs. It would be interesting to continue the development of this serological test against other streptococci, coagulase-negative staphylococci and Gram-negative bacilli such as *Enterobacteriaceae*.

One of the major issues for the coming years would be to diagnose PJI as early as possible before or during revision surgery to guide the type and conditions of the surgical revision. A panel of tests has recently become available for the diagnosis of PJI. Among them, 2 (the leukocyte esterase test strip and the alpha-defensin test) can be performed directly from synovial fluid, even during the surgical procedure 13-19. Both have been the subject of numerous studies. In a systematic review and meta-analysis, Wyatt et al. 12 showed a high diagnostic accuracy for PJI for both tests, despite substantial heterogeneity among studies. Comparing with frozen section histology, the leukocyte esterase test strip was found to be faster and easier with low costs and has been proposed as a valuable alternative to frozen section histology 13. To date, some systematic reviews and meta-analyses demonstrated high pooled sensitivity and specificity of the synovial alpha-defensin biomarker 14-16. The alpha-defensin quick on-table lateral flow test (Synovasure, Zimmer Biomet, USA), proposing maximum specificity in a short time, has even been proposed as a perioperative confirmatory test for PJI 17. However, some other studies should moderate the recent enthusiasm for these biomarkers, now considered as minor diagnostic criteria for PJI 6,8. Kleist et al. 18 recently showed that the performances of the synovial fluid alpha-defensin ELISA are insufficient to accurately diagnose PJI, especially due to coagulase-negative staphylococci 18. Two recent studies concluded that the performance of the alpha-defensin lateral flow test was inferior to that of the alpha-defensin ELISA 19,20. Finally, some authors suggest the risk of false-positive results both with the leukocyte esterase test and alpha-defensin test in patients with a diagnosis of metal-on-metal failure 20-22.

These contradictory results encourage us to be cautious. Moreover, biomarker tests do not provide information on the identity of the infectious pathogen. In all cases, other diagnostic tools deserve to be evaluated and preoperative serological diagnosis is one of them.

There is no doubt that the performance of the BJI InoPlex® serological test remains, in this first version, insufficient for the diagnosis of PJI caused by *S. aureus*, *S. epidermidis* and *S. lugdunensis*. The interest in the test may be underlined when cultures remain sterile in treated patients. The PPV of a positive serological test for targeted staphylococci in association with a CRP >10 mg/L was 85.71%. The excellent negative predictive value obtained for all the bacteria most often isolated in PJIs represents a real interest. Certainly, a negative CRP level has an NPV at least equal to 90% and is easier and less expensive than the serological test. However, the absence of detection of anti-staphylococcal antibodies could be useful in choosing a probabilistic treatment that does not include an anti-staphylococcal molecule.

## Figures and Tables

**Table 1:** Baseline characteristics of the study population

	Total (N = 115)	Uninfected (n = 66)	Infected (n = 49)	*p* value
Age (years), median (IQR)	69 (63-78)	71 (63-78)	68 (62-79)	0.969
Male sex	57 (49.6)	29 (43.9)	28 (57.1)	0.161
Immunosuppressive therapy	8/114 (7)	4/65 (6.2)	4/49 (8.2)	0.678
Rheumatoid arthritis	4/114 (3.5)	2/65 (3.1)	2/49 (4.1)	0.773
Recent or ongoing antibiotic therapy	11/113 (9.7)	3/65 (4.6)	8/48 (16.7)	0.033
Hospitalization within 3 months	14/106 (13.2)	3/62 (4.8)	11/44 (25.0)	0.003
Site of prosthesis				
Hip	77 (67.0)	47 (71.2)	30 (61.2)	0.300
Knee	34 (29.6)	18 (27.3)	16 (32.6)	
Shoulder	4 (3.5)	1 (1.5)	3 (6.1)	
Time since insertion of the prosthesis (years), median (IQR)	8 (3-18)	13 (4-21)	6 (2-10)	0.001
Sinus tract	16 (13.9)	0	16 (32.6)	<0.0001
CRP ≥10 mg/L^a^	45/111 (40.5)	10/64 (15.6)	35/47 (74.5)	<0.0001
CRP (mg/L), median (IQR)	6 (1-19)	2.7 (1-6.6)	19 (7.8-32.4)	<0.0001

Values are number (%) except where indicated otherwise. IQR, interquartile range; ^a^Missing data for 4 patients; total population 111.

**Table 2:** Microbial species involved in the preoperative or perioperative samples

Documentation of bacterial PJI	All sites	Hip	Knee	Shoulder
Monomicrobial sepsis, n = 43				
*Staphylococcus aureus*	13^a^	9	3	1
*Staphylococcus epidermidis*	10	5	3	1
*Staphylococcus lugdunensis*	1	1	0	0
Other coagulase-negative staphylococci^b^	8	4	4	0
*Streptococcus agalactiae*	1	1	0	0
*Streptococcus/Enterococcus* spp^c^	5	4	1	0
*Cutibacterium acnes*	2	1	1	0
*Corynebacterium striatum*	1	1	0	0
*Pseudomonas aeruginosa*	1	1	0	0
*Mycobacterium bovis*	1	0	1	0
Polymicrobial sepsis (n = 6)				
*S. aureus*, *S. epidermidis*, *C. acnes*	1	0	0	1
*S. lugdunensis*, *Finegoldia magna*	1	0	1	0
*S. aureus*, *Streptococcus dysgalactiae*	1	1	0	0
*S. epidermidis*, *F. magna*	1	0	1	0
*S. capitis*, *Enterococcus faecalis*	1	1	0	0
*Enterobacter cloacae*, *S. dysgalactiae*	1	0	1	0
Total	49	30	16	3

^a^PCR 16S positive for *S. aureus*, n = 1.^b^*S. capitis*, n = 5; *S. warneri*, n = 2; *S. saprophyticus*, n = 1. ^c^*S. dysgalactiae*, n = 1; *S. mitis*/*oralis*, n = 1; *S. mutans*, n = 1; *E. faecalis*, n = 2.

**Table 3:** Performances of serological tests according to cultures

Bacterial species	Infected patients (n = 49)		Uninfected patients (n = 66)		Sensitivity (%)	Specificity (%)	PPV (%)	NPV (%)
	TP	FN	TN	FP				
Targeted Staphylococci (n=112)^a^	21	7	69	15	75 (21/28)	82.1 (69/84)	58.3 (21/36)	90.8 (69/76)
Streptococcus agalactiae (n=112)^a^	1	0	108	3	NA	97.3 (108/111)	NA	100 (108/108)
Cutibacterium acnes (n=114)^a^	0	3	93	18	NA	83.8 (93/111)	NA	96.9 (93/96)

PPV, positive predictive value; NPV, negative predictive value; TP, true positive; FP, false positive; TN, true negative; FN, false negative. NA, not available. ^a^One indeterminate result excluded from the analysis.
